# Evaluation of the anti-inflammatory, antioxidant and regenerative effects of microbiota-derived postbiotics in human periodontal ligament mesenchymal stromal cells

**DOI:** 10.1007/s00784-025-06341-1

**Published:** 2025-04-23

**Authors:** Hazal Kibar Demirhan, Emine Omer Oglou, Zeynep Busra Aksoy, Fadime Kiran

**Affiliations:** 1 Pharmabiotic Technologies Research Laboratory, Department of Biology, Faculty of Science, Ankara University, Ankara, 06100 Turkey; 2 Graduate School of Natural and Applied Sciences, Ankara University, Ankara, 06110 Turkey; 3 Stem Cell Institute, Ankara University, Ankara, 06520 Turkey

**Keywords:** Periodontal disease, Postbiotics, Regeneration, Inflammation, Migration, Oxidative damage

## Abstract

**Objective:**

This study investigates the regenerative and protective effects of postbiotics (cell-free supernatant) derived from the *Lactiplantibacillus plantarum* EIR/IF-1 strain on human periodontal ligament mesenchymal stromal cells (hPDL-MSCs).

**Materials and methods:**

hPDL-MSCs were isolated from periodontal ligament tissues (PDL) of wisdom teeth using enzymatic digestion and subsequently characterized through immunophenotyping. The effect of postbiotics on the viability of hPDL-MSCs was assessed using the MTT assay and flow cytometry, while their impact on cell migration was evaluated via the scratch assay. Anti-inflammatory effects of postbiotics were investigated on lipopolysaccharide (LPS, derived from *Porphyromonas gingivalis*)-stimulated hPDL-MSCs through Enzyme-Linked Immunosorbent Assay (ELISA). Additionally, the antioxidant effects of postbiotics were analyzed in hydrogen peroxide (H₂O₂)-induced hPDL-MSCs by measuring reactive oxygen species (ROS) levels using flow cytometry. The expression of collagen type I (*COL1A1*) gene was further assessed by quantitative reverse transcription PCR and immunofluorescence staining.

**Results:**

Treatment with postbiotics (250 µg/mL) significantly increased the viability and migration capability of hPDL-MSCs, while enhancing collagen production for PDL repair. Treatment with postbiotics for 24 h resulted in a 54.53 ± 2.01% reduction in intracellular ROS levels compared to untreated H_2_O_2_-induced hPDL-MSCs. Furthermore, postbiotics significantly decreased the production of pro-inflammatory cytokines (IL-8, IL-6, and IL-1β), and increased the anti-inflammatory cytokine IL-10 (2.67-fold) compared to untreated LPS-stimulated hPDL-MSCs.

**Conclusion:**

Our findings indicate that postbiotics exhibit biological activity throughout all stages of the healing process, beginning with the modulation of the inflammatory response to LPS stimulation, followed by the promotion of cell migration, proliferation, and collagen synthesis. Given the unmet need for safe and adjuvant therapeutic approaches that promote comprehensive periodontal regeneration in periodontal diseases, this study presents postbiotics as a promising candidate.

**Clinical relevance:**

Postbiotics could be integrated into regenerative therapies as a novel bioactive material to improve the healing and regenerative outcomes in periodontal defects by both controlling inflammation and stimulating tissue repair processes.

**Supplementary Information:**

The online version contains supplementary material available at 10.1007/s00784-025-06341-1.

## Introduction

Periodontal diseases, such as gingivitis and periodontitis, are one of the most widespread oral health conditions globally, with a prevalence reaching up to 50% worldwide [1]. Gingivitis represents the initial and mildest form of the disease. However, if neglected and untreated, it can progress, leading to further degenerative changes in the tissues and ultimately resulting in periodontitis [2]. As an inflammatory condition driven by biofilm-induced dysbiosis, periodontitis has emerged as a significant public health concern [3] affecting approximately 1.1 billion individuals worldwide, which represents 13.1% of the global population [4]. To date, numerous studies [1, 5, 6] have highlighted the consequences of periodontitis, including the destruction of soft tissues such as gingiva, periodontal ligament (PDL), and periodontal pocket, as well as the resorption of hard tissues (e.g., marginal alveolar bone loss), that may ultimately lead to tooth loss. Although current conventional periodontal therapies (improving oral hygiene, dental biofilm control, mechanical removal of plaque, local or systemic antibiotic therapy) focus on biofilm removal that mitigates the inflammation and aggressive progression of periodontitis, they cannot achieve complete periodontal regeneration which involves the formation of new cementum, alveolar bone, and functional PDL on the periodontitis root surface [7–9]. In light of the challenges associated with treatment of periodontitis, there is an urgent need for alternative therapeutic strategies to achieve complete periodontal regeneration [10].

For years, mesenchymal stromal cells (MSCs) have been investigated as a promising therapeutic strategy, not only for tissue regeneration [11], but also for immunotherapy [12] and chronic diseases [13]. Recently, dental MSCs, isolated from various sources within the oral cavity, have emerged as a promising approach for inducing functional dental tissue regeneration [10]. Among these, human periodontal ligament mesenchymal stromal cells (hPDL-MSCs) are particularly noteworthy for their role in maintaining periodontal tissue homeostasis and promoting regeneration, particularly in the context of periodontitis. Their high therapeutic potential is likely attributed to their immunomodulatory properties including the suppression of peripheral blood mononuclear cell and CD4 + T cell proliferation, as well as the promotion of macrophage polarization towards an anti-inflammatory M2 phenotype. However, the inflammatory environment can modulate each of these mechanisms [14, 15]. Given their promising therapeutic potential, the number of clinical trials exploring the use of hPDL-MSCs, either in combination with scaffolds, membranes, or signaling molecules, or as standalone therapies, has been steadily increasing in the field of regenerative periodontology [16]. Despite the development of various therapeutic approaches utilizing hPDL-MSCs, their application in contemporary clinical practice for repairing the affected periodontium remains a significant challenge. This is primarily due to factors such as the limited availability of cells and the lack of multifunctional efficacy in treatment strategies that address different stages of periodontitis. To overcome these challenges, natural compounds have been proposed to enhance the survival, biological function, and therapeutic potential of hPDL-MSCs [17].

Over the past few decades, research has elucidated the pivotal role of the oral microbiota in health and disease [18]. Recently, there has been growing interest in the use of probiotics as a novel approach to prevent and treat periodontal disease [19]. The mechanisms in which probiotics act in the prevention and treatment of periodontal disease involve inhibiting the growth and virulence of pathogenic species and restoring microbial balance in the oral microbiota, modulating the immune response, and promoting tissue healing processes [20]. The available forms of probiotics for dental diseases include oral probiotic lozenges, gums, toothpaste, mouthwashes, and supplements [21]. Despite their strong impact on the oral health, conventional therapies based on active probiotics often face significant commercial and technological challenges related to the maintenance of their cellular viability during the production, storage, or delivery of the final product [22]. Moreover, safety concerns regarding the overuse of probiotics in immunocompromised patients and infants have also attracted attention [23]. To overcome these disadvantages, the application of metabolites or fragments derived from probiotics (postbiotics) has garnered recent interest as a novel therapeutic and preventive strategy in modern medicine. In mid-2021, the International Scientific Association for Probiotics and Prebiotics (ISAPP) defined the postbiotic as, “a preparation of inanimate microorganisms and/or their components that confers a health benefit on the host” [24]. Based on this definition, postbiotics can contain microbial cell fractions, cell lysates, short-chain fatty acids (SCFAs), extracellular polysaccharides (EPS), teichoic acid, organic acid, proteins, enzymes, and muropeptides derived from peptidoglycan. Since postbiotics do not contain live cells, the clinical application of postbiotics could offer an alternative with enhanced safety, stability, and efficacy, especially in cases where live microorganisms may pose challenges or risks. Importantly, they maintain beneficial effects through similar mechanisms to those of probiotics. Therefore, postbiotics have recently attracted interest in novel therapeutic strategies with their broad-scale modulatory effects including immunomodulatory, antimicrobial, anti-inflammatory, antioxidant, and proliferative properties [25]. These attributes render postbiotics a promising therapeutic strategy particularly in the context of periodontal health and regeneration [26]. Nevertheless, the selection of the ideal postbiotic-producer strains requires special attention depending on the target case because of that their efficacy largely depends on the bacterial strain. Consequently, recent studies have focused on discovering novel strains with multi-functional properties and their incorporation into next-generation dental products. While probiotics have their own unique benefits, the clinical application of postbiotics could offer an alternative with enhanced safety, stability, and efficacy, especially in cases where live microorganisms may pose challenges or risks.

This study aims to evaluate the regenerative, antioxidant, and anti-inflammatory effects of postbiotics derived from the *Lactiplantibacillus plantarum* EIR/IF-1 strain, previously isolated from the fecal microbiota of a breastfed infant, on hPDL-MSCs. This strain was selected for its demonstrated antimicrobial, anti-biofilm and anti-quorum sensing properties, particularly against pathogens implicated in dental diseases (*Filifactor alocis*, *Fusobacterium nucleatum*, *Porphyromonas gingivalis*, *Prevotella denticola*, *Streptococcus mutans*, and *Streptococcus sanguinis*) [27–29]. The observed effects of postbiotics on periodontitis-associated pathogens highlight their potential for integration into therapeutic strategies. To further strengthen our hypothesis, this study includes analyses investigating their role in tissue regeneration, as well as their capacity to mitigate oxidative damage and inflammation. Our findings not only advance our understanding of the effects of postbiotics on periodontal tissues but also introduce innovative approaches with the potential to revolutionize dental therapies and reshape the future of periodontal treatments.

## Materials and methods

### Bacterial strain and culture conditions

The *Lactiplantibacillus plantarum* EIR/IF-1 strain (NCBI GenBank accession number: OP810909.1), previously isolated and identified, was used in this study as a postbiotic producer. This strain was obtained from the Culture Collection of the Pharmabiotic Technologies Research Laboratory, Department of Biology, Ankara University (Ankara, Turkey), and preserved at -80 °C in de Man, Rogosa, and Sharpe (MRS; Merck, Germany) broth supplemented with glycerol (50:50%, v/v). Prior to use, the strain was cultured in MRS broth at 37 °C under static aerobic conditions for 24 h.

### Preparation of postbiotics (cell-free supernatant)

The bacterial strain (100 µL) was cultured in 100 mL of MRS broth at 37 °C for 24 h. After incubation, the supernatant of bacterial culture was obtained by centrifugation at 15.000×*g* for 20 min, and sterile filtration was performed using a membrane filter with a pore size of 0.22 μm (Sartorius, Germany) to completely remove the cells. The filtered supernatant was subsequently lyophilized under freezing conditions of -20 °C, vacuum pressure of 0.120 mB, and a condenser temperature of -58 °C, using a freeze dryer (Buchi, Switzerland) for 48 h. The resulting lyophilized powder was dissolved in distilled water (1 g/mL), sterilized through membrane filtration as previously described, and stored at -20 °C for subsequent assays [27]. For the purpose of standardization, identical conditions were maintained across all batches.

### Isolation of human periodontal ligament mesenchymal stromal cells

**Fig. 1:**
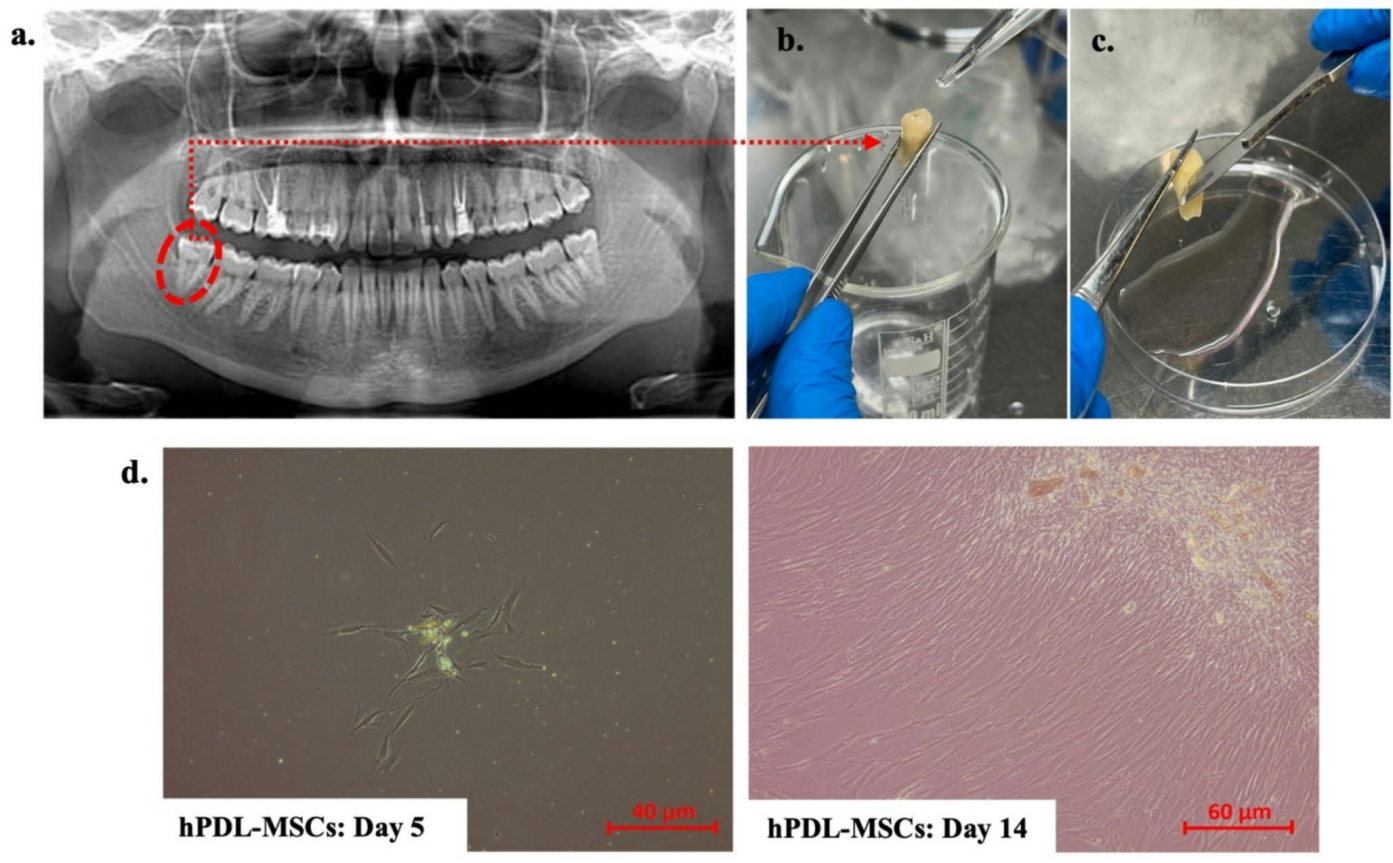
Isolation of human periodontal ligament mesenchymal stromal cells (hPDL-MSCs). (**a**) Panoramic radiograph of the patient, (**b**) Washing the extracted teeth with1× phosphate-buffered saline, (**c**) Extracting the periodontal ligament tissue, (**d**) Micrographs of cells observed under inverted microscope on day 5 and day 14 after seeding

Human periodontal ligament mesenchymal stromal cells (hPDL-MSCs) were isolated from periodontal ligament (PDL) tissues attached to one-third of the roots of wisdom teeth from four volunteers (two males and two females), aged 20–40 years, who were undergoing orthodontic treatment (Fig. 1a). All experimental protocols were approved by the Ethics Committee of Karamanoglu Mehmetbey University, Karaman, Turkey (Protocol code: 01-2023/20). Briefly, four healthy human teeth were collected in a container containing Dulbecco’s phosphate buffered saline (DPBS) supplemented with 1% antibiotic antimycotic (Gibco, Thermo Fisher Scientific, USA) under aseptic conditions and immediately transferred to the laboratory. The extracted teeth were then washed three to five times using 1× phosphate-buffered saline (PBS, Sartorius, Germany) solution in a sterile petri dish (Fig. 1b). The middle-third of the roots were scraped gently using a Bard-Parker’s blade number 15 into a sterile petri-dish and segmented into pieces of 1–3 mm size (Fig. 1c). The collected PDL tissues were digested in a solution of 3 mg/mL collagenase Type I (Gibco, UK) and 4 mg/mL dispase (Gibco, UK) for 1 h at 37 °C. The mixture was then transferred through a 70 μm pore size cell strainer (BD Falcon Labware, Franklin Lakes, NJ, USA) to obtain single-cell suspension. The resulting cells were subsequently pelleted by centrifugation at 1.800 rpm and cultured in a 25 cm² filtered flask (Sarstedt, Germany) containing alpha modified of Eagle’s medium (α-MEM Sigma-Aldrich, USA) supplemented with 15% fetal bovine serum (FBS; Gibco, UK), 2mM L-glutamine (Gibco, UK), and 100 U/mL penicillin/100 mg/mL streptomycin (Gibco, UK) for 7 days in a humidified atmosphere of 5% carbon dioxide (CO₂) at 37 °C, reaching 70–80% confluency under standard culture conditions. The medium was refreshed every 3–4 days. Once the cells reached 80% confluency, they were rinsed with PBS, harvested with 0.25% trypsin-EDTA (Sartorius, Germany), and centrifuged at 200×*g* for 5 min. The cells were stained with trypan blue (Sartorius, Germany) and counted using a TC20 Automated Cell Counter (Bio-Rad, USA) for further assays [30]. Experiments were performed using either passage (P) 4 or 5, and the cells were regularly tested for bacteria, fungi, and mycoplasma contamination using commercial PCR-based kit protocols (Thermo Scientific, USA).

### Flow cytometric analysis of hPDL-MSCs phenotype

To verify the identity of the isolated cells, expression of surface markers was analyzed using flow cytometry. Cells were cultured in 25 cm^2^ polystyrene cell culture flasks in αMEM, washed twice carefully in PBS, detached from the culture flasks using 0.25% trypsin-EDTA, and subsequently centrifuged at 200x*g* for 5 min at 4 °C. Harvested cells were fixed with 4% paraformaldehyde for 30 min at 4 °C, and then permeabilized with 0.1% Triton X-100 for 10 min at room temperature, and blocked with a solution of 0.5% bovine serum albumin (BSA) and 0.02% Tween-20 for 20 min at room temperature. Next, the cells were incubated with primary monoclonal antibodies raised against CD31, CD34, CD45, CD73, CD90, and CD105 according to the standard quantities recommended by the manufacturer (Thermo Fisher Scientific, USA), at 37 °C for 1 h in the dark. After incubation, the cells were washed twice with PBS, and treated with fluorescent secondary antibody for 40 min at 4 °C in the dark [30]. The cells were then analyzed by flow cytometry (NovoCyte, ACEA Biosciences, USA) using FlowJo software (version 10.5.3, Tree Star, San Carlos, USA). Unstained cells served as negative control were used to establish gating parameters.

### Cell viability assays

An MTT assay (3-(4,5-dimethylthiazol-2-yl)-2,5-diphenyltetrazolium bromide) was conducted to evaluate the cytotoxic effects of postbiotics on hPDL-MSCs [31]. Briefly, 1 × 10^4^ cells per well were seeded into 96-well cell culture plates (Sarstedt, Germany) and allowed to adhere for 24 h under standard culture conditions (37 °C with 5% CO_2_ and 95% relative humidity). Subsequently, the culture medium was replaced with fresh α-MEM containing different concentrations of postbiotics, ranging from 2.5 to 1000 µg/mL. Untreated cells were used as negative controls. Following 24 h of incubation, 10 µL of MTT solution (5 mg/mL, Sigma-Aldrich, USA) was added to each well, and the plates were incubated in darkness for 4 h. The formazan crystals formed by the viable cells were then solubilized in 100 µL of dimethyl sulfoxide (DMSO, Serva, USA). Finally, the optical density of each well was measured at 570 nm using a microplate reader (Epoch, USA).

The Annexin V-FITC Apoptosis Detection Kit (BioLegend, USA) was employed to quantitatively assess cell viability via flow cytometry, following the manufacturer’s instructions. A total of 1 × 10^5^ cells per well were seeded into 6-well cell culture plates (Sarstedt, Germany) and treated with postbiotics for 24 h, as previously described. Untreated cells were used as negative controls. After incubation, the cells were stained with FITC-conjugated Annexin V and propidium iodide (PI) and subsequently analyzed using a flow cytometer. Data analysis was performed using FlowJo software.

### Migration assay

The impact of postbiotics on the motility of hPDL-MSCs was assessed by using a scratch assay, as described by Liang et al. [32]. Briefly, cells were seeded in 6-well cell culture plates at a density of 1 × 10^5^ cells per well and incubated under standard conditions until a confluent monolayer was formed. A sterile pipette tip was used to create a scratch across the center of each well, and cellular debris was removed by gently washing the cells twice with PBS. The cells were subsequently incubated in a fresh medium supplemented with postbiotics at the highest non-cyotoxic doses, as determined by the cell viability assay, while untreated cells served as controls. Cell migration into the wound area was monitored every 3 h using an inverted microscope (Olympus, Japan). The distance between the two edges of the wound area was measured using ImageJ software (NIH, USA), and the percentage of wound closure was calculated using the equation: (At_0_ - At/At_0_) x 100, where At_0_ is represents the scratch area at 0 h and At represents the corresponding scratch area at different time intervals.

### Determination of intracellular reactive oxygen species (ROS) levels

Intracellular ROS levels were quantified by flow cytometry analysis. Hydrogen peroxide (H₂O₂) was employed to induce ROS production in hPDL-MSCs. Initially, the effects of varying H₂O₂ concentrations on hPDL-MSCs viability were determined using the MTT assay. The H₂O₂ concentration that reduced cell viability by 50% (IC₅₀) after 24 h of exposure and led to significant ROS accumulation was selected for subsequent treatments. Intracellular ROS levels in H₂O₂-challenged hPDL-MSCs, treated with or without postbiotics for 24 h, were evaluated using the Cellular ROS Assay Kit (Abcam, UK) according to the manufacturer’s instructions. Briefly, cells were seeded in 6-well cell culture plates at a density of 1 × 10^5^ cells per well and treated with postbiotics for 24 h under standard conditions. Untreated cells were used as negative controls. After treatment, cells were trypsinized, centrifuged, and resuspended in 100 µL of 25 µM 2,7-dichlorodihydrofluorescein diacetate (DCFH-DA) solution, a cell-permeable ROS-sensitive fluorescent probe. The suspension was incubated at 37 °C for 30 min in darkness. ROS accumulation was subsequently measured at an excitation wavelength of 485 nm and an emission wavelength of 528 nm using a fluorescence multi-detection reader integrated with flow cytometry. Data analysis was performed using FlowJo software.

### Measurement of immunological markers

The effect of postbiotics on cytokine production in hPDL-MSCs was assessed following lipopolysaccharide (LPS)-induced inflammation. LPS derived from *P. gingivalis* (MQ300, Sigma-Aldrich, USA) was used as the inflammatory agent. Initially, hPDL-MSCs cells were cultured in α-MEM medium with or without different concentrations of *P. gingivalis* LPS (0 µg/mL, 1 µg/mL, 5 µg/mL, and 10 µg/mL) for 24 h [33]. The lowest non-cytotoxic dose of LPS was determined via MTT assay as described previously. hPDL-MSCs were then seeded at a density of 1 × 10^4^ cells per well in 96-well cell culture plates and treated with the non-cytotoxic concentration of LPS, either alone or in combination with postbiotics. Following a 24 h incubation period, cytokine levels, including interleukin (IL)-10, IL-8, IL-6, and IL-1β, were quantified in the cell culture supernatants using Enzyme-Linked Immunosorbent Assay (ELISA) kits (MabTech, Switzerland), in accordance with the manufacturer’s protocols.

### Analysis of collagen deposition

The expression of collagen type I (*COL1A1*) gene was assessed using quantitative reverse transcription PCR (qRT-PCR) and immunofluorescence staining. For qRT-PCR, total RNA was extracted from hPDL-MSCs (1 × 10^6^ cells per well) treated with or without postbiotics for 24 h, using PureZOL™ reagent (Bio-Rad, Hercules, CA, USA) according to the manufacturer’s protocol. Following RNA extraction, complementary DNA (cDNA) was synthesized using the iScript™ cDNA Synthesis Kit (Bio-Rad, USA). qRT-PCR was performed on a Bio-Rad CFX96 Touch Real-Time PCR system using specific primers designed for the genes of *COL1A1* (F:5’-CCCTCCACTCCTTCCCAAAT-3’; R:5-CGAAGGGGAGATGTTGCAAG-3’) and *β-actin* (F:5’- GCTCTTTTCCAGCCTTCCTT-3’; R:5’-CAGCAATGCCAGGGTACATG-3’) as an internal control, using Primer3 software and the NCBI-Primer BLAST program. The PCR reaction mixture included 5 µL of 1x SYBR Green Master Mix (Bio-Rad, USA), 0.5 µL of forward and reverse primers (0.5 mM each), and 1 µL of cDNA. Amplification was carried out under the following conditions: initial denaturation at 95 °C for 3 min, followed by 40 cycles at 95 °C for 10 s, annealing at 60 °C for 30 s, and DNA strand synthesis at 72 °C for 30 s. The relative quantification of target gene expression was expressed as threshold cycle numbers (Ct) and normalized to internal controls. The fold change in gene expression was quantified using the 2^−ΔΔCt^ method, where ΔCT represents the difference between the mean CT values of triplicate samples.

Immunofluorescence detection of COL1A1 was performed using fluorescence microscopy. Briefly, hPDL-MSCs (1 × 10^6^ cells/mL) grown on glass coverslips with or without postbiotics for 24 h were fixed in 4% paraformaldehyde (PFA, Sigma-Aldrich, USA) in PBS for 20 min. The fixed cells were subsequently treated with a primary antibody against COL1A1 (collagen type I). This was followed by the application of a secondary antibody conjugated with FITC (Fluorescein isothiocyanate). Nuclear DNA was stained using 4’,6-diamidino-2-phenylindole (DAPI, Thermo Fisher Scientific, USA), which emits blue fluorescence for cellular nuclei visualization. Slides were then mounted in ProLong Gold Antifade Reagent (Invitrogen, USA) and examined using a fluorescence microscope (Olympus BX60) equipped with a Color View III cooled CCD camera. The excitation wavelength was set to 488 nm, and the emission wavelength was monitored at 520 nm for capturing fluorescence signals.

### Data analysis

All assays were conducted across three independent experiments, with each measurement performed in triplicate. Data analysis was carried out using GraphPad Prism software version 8.0.2 (San Diego, CA, USA). The student’s t-test was employed to compare the means between two groups, while analysis of variance (ANOVA) was utilized to compare the means across three or more groups. All results were presented as the mean ± standard deviation and *p*-values less than 0.05 were considered to indicate a statistically significant difference.

## Results

### Characteristics of hPDL-MSCs

hPDL-MSCs were isolated from wisdom teeth using enzymatic digestion and cultured in the α-MEM. Initially, the cells appeared small and thin by day 3, exhibiting a spindle-shaped morphology characteristic of fibroblast-like cells by day 5. By day 14, the fibroblast-like cells reached approximately 80% confluence (Fig. 1d). The hPDL-MSCs were further characterized through cell surface marker analysis. Flow cytometry results revealed that hPDL-MSCs expressed mesenchymal stem cell-associated markers, including CD73 (95.90%), CD90 (95.80%), and CD105 (94.81%), while lacking expression of hematopoietic markers such as CD34 (2.72%) and CD45 (2.19%), as well as the endothelial marker CD31 (0.65%) (Supp. Figure 1).

### Proliferative effects

**Fig. 2:**
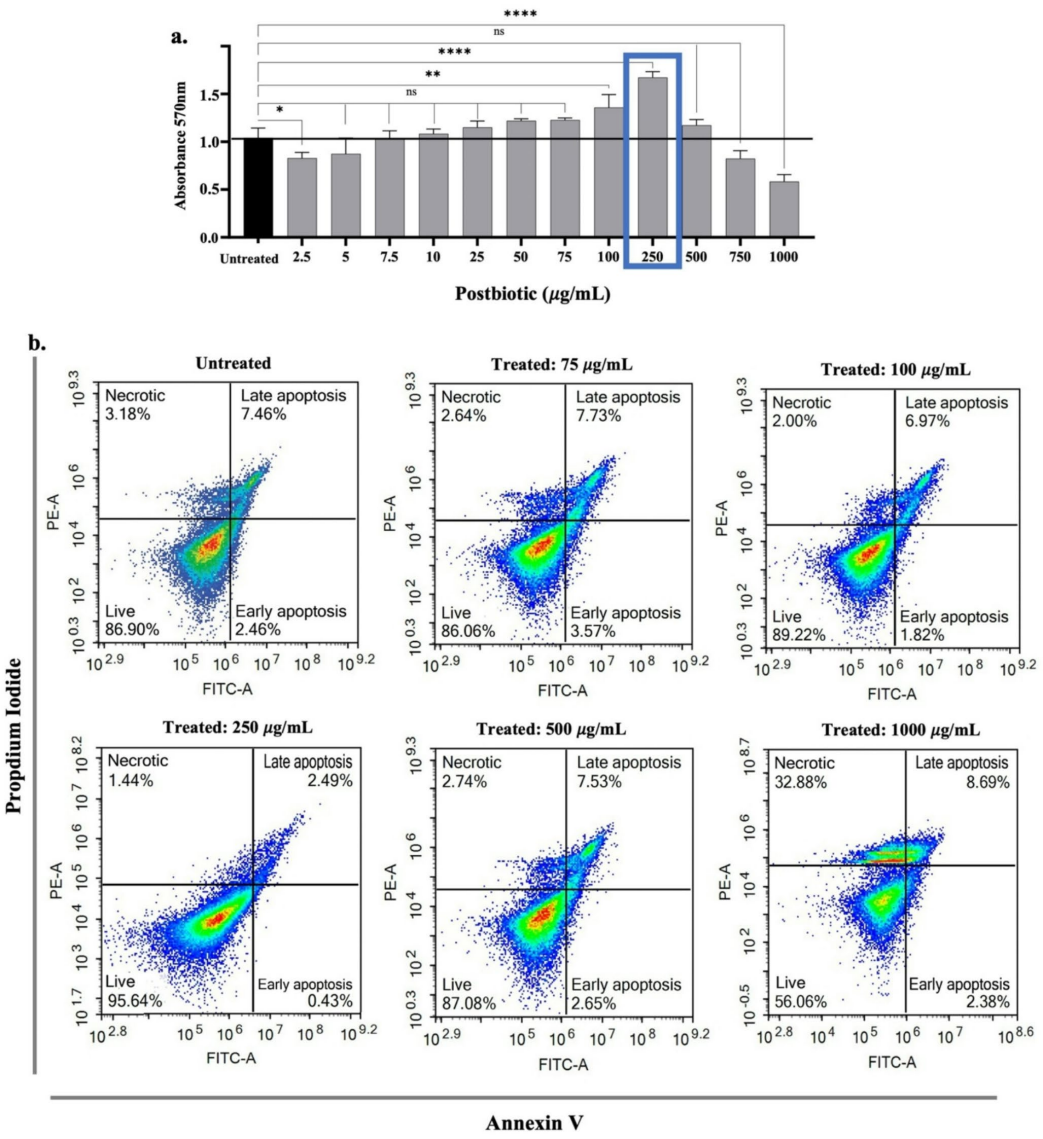
Cell viability of hPDL-MSCs following postbiotic treatment, assessed by (**a**) MTT assay and (**b**) flow cytometry analysis using Annexin V-FITC/PI staining. The flow cytometry quadrants represent necrotic cells (PI-positive only), late apoptotic cells (annexin V and PI double-positive), early apoptotic cells (Annexin V-positive only), and live cells (non-apoptotic cells). Data are expressed as mean ± SD (*n* = 3). Statistical significance is denoted as follows: * *p* < 0.05, ** *p* < 0.01, **** *p* < 0.0001, n.s: non-significant

To evaluate the effects of postbiotics on the viability of hPDL-MSCs, an MTT assay was performed. Increasing doses of postbiotics resulted in a plateau effect, with the highest cell viability observed at a concentration of 250 µg/mL (*p* < 0.0001). No significant effects were noted at doses ranging from 5 to 75 µg/mL and 500–750 µg/mL. However, a significant increase in cell viability was also observed in 100 µg/mL (*p* < 0.01). In contrast, treatment with 1000 µg/mL led to a significant decrease in cell viability (*p* < 0.0001) (Fig. 2a). Since the MTT assay only provides data on cell viability or cytotoxicity at a single time point, additional experiments were conducted using Annexin V-FITC/PI staining to gain a more comprehensive understanding of the proliferative effects of postbiotics on hPDL-MSCs. The flow cytometry results confirmed the findings from the MTT assay. Compared to untreated cells (86.90% cell viability), a significant increase in the proportion of live hPDL-MSCs (95.64%) was observed following the treatment with 250 µg/mL of postbiotics (*p* < 0.05), as indicated by the lower left quadrant of the flow cytometry plots, representing viable cells negative for Annexin-FITC and PI. Furthermore, a significant reduction (*p* < 0.001) in the proportion of live cells (56.06%) was observed when treated with 1000 µg/mL of postbiotics (Fig. 2b). Based on these findings, the 250 µg/mL postbiotic concentration which exhibited the most pronounced effects on hPDL-MSCs, was selected for further assays.

### Induction of migration

**Fig. 3:**
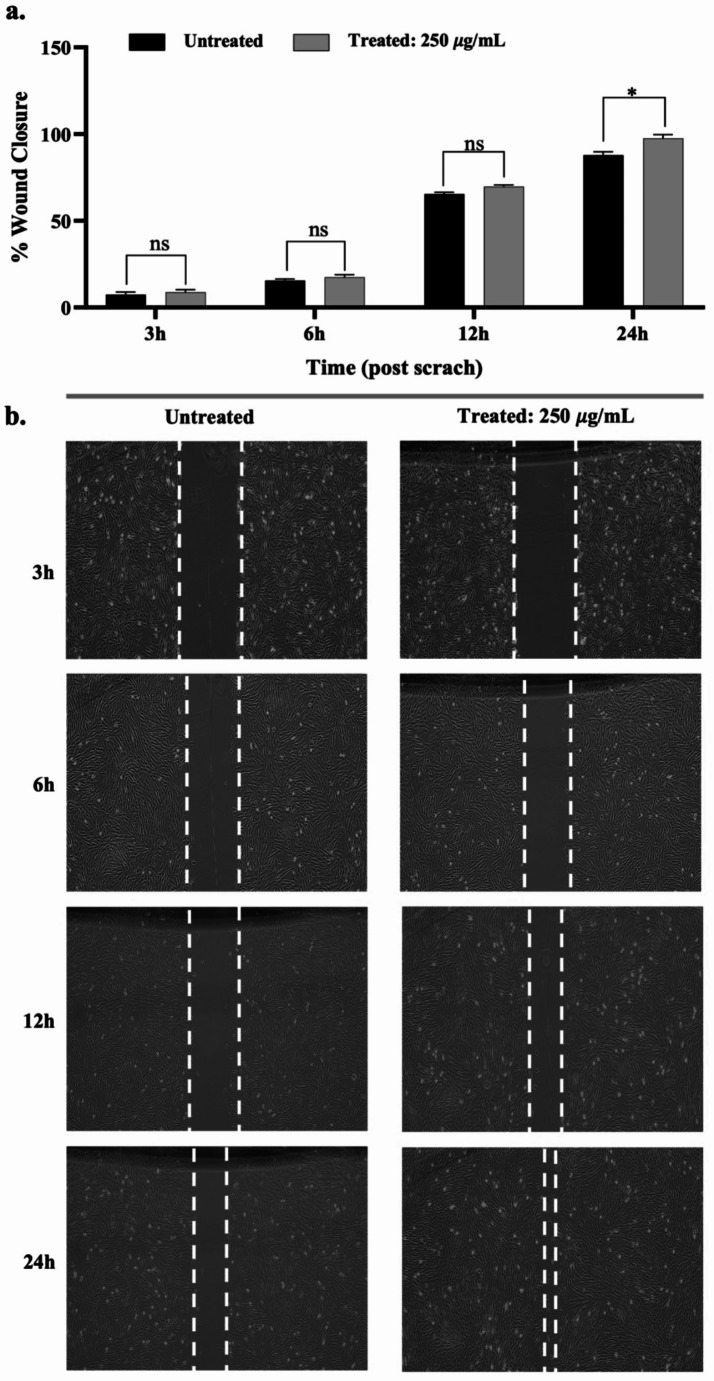
Effects of postbiotics on the migration capability of hPDL-MSCs. (**a**) Wound closure (%) at different time points post-injury, (**b**) Representative photomicrographs of hPDL-MSCs during scratch assay at the indicated time points, captured using a 10x objective on an inverted microscope. Data are expressed as mean ± SD (*n* = 3). Statistical significance is denoted as follows: * *p* < 0.05, n.s: non-significant

The effect of postbiotics (250 µg/mL) on the migration ability of hPDL-MSCs was assessed using a scratch assay. At 24 h post-injury, the wound area was reduced to 6.86 × 10^8^± 0.28 µm^2^, corresponding to a significant relative wound closure of 97.61 ± 4.71% (*p* < 0.05) compared to the control. In addition, untreated cells achieved 82.09 ± 3.54% wound closure (Fig. 3a). Microscopic observation further revealed that the scratch wound in the treated wells was significantly filled with cells, which was attributed to accelerated cell proliferation compared to the untreated samples (Fig. 3b), after 24 h of treatment.

### Reduction of ROS accumulation

**Fig. 4:**
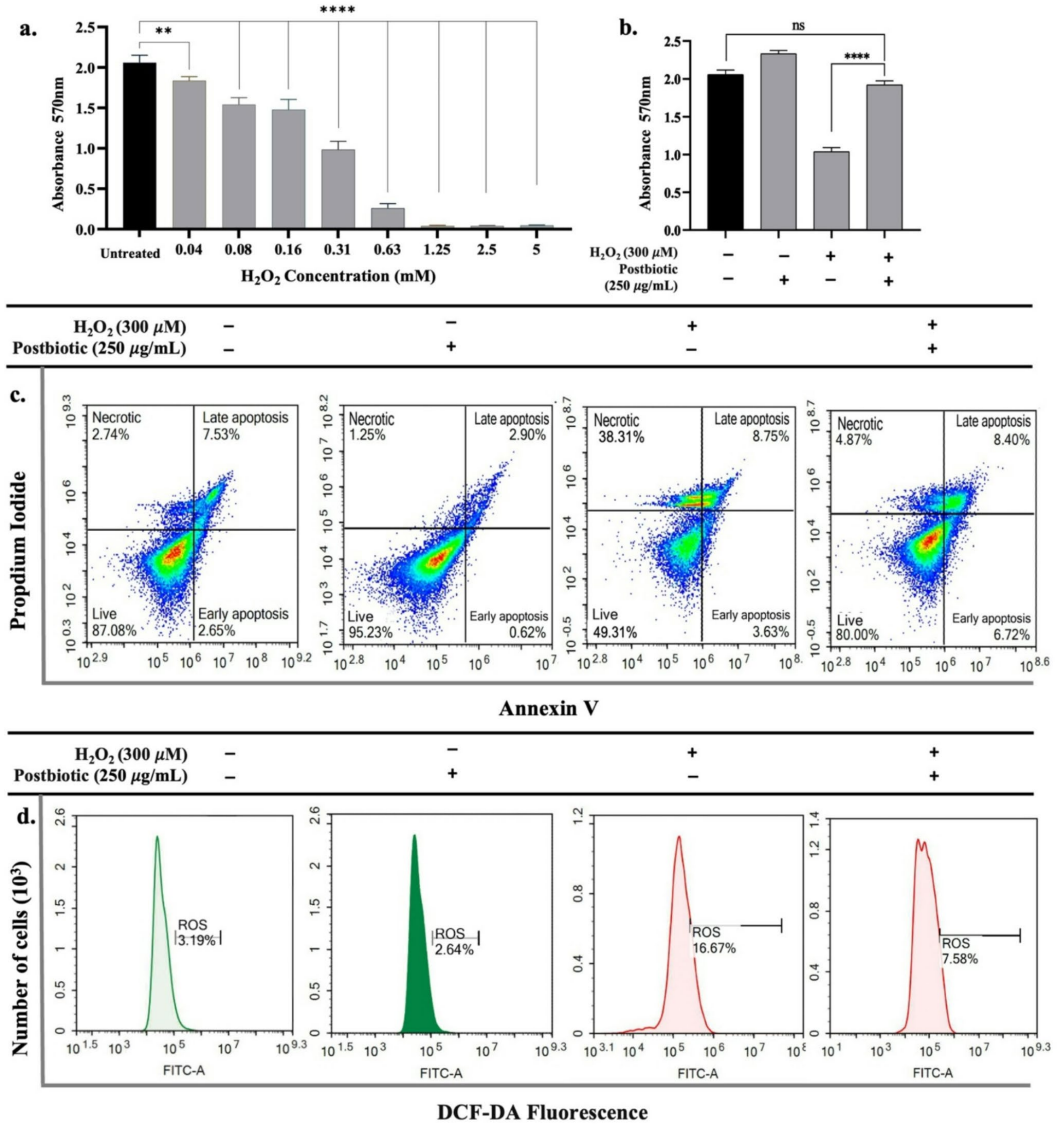
Effects of postbiotics on the H_2_O_2_-induced ROS levels in hPDL-MSCs. (**a**) Cell viability of hPDL-MSCs following treatment with various concentrations of H_2_O_2_, (**b**) Viability of hPDL-MSCs treated with selected doses of H_2_O_2_ with or without postbiotics, as determined by MTT assay and (**c**) Flow cytometry analysis using Annexin V-FITC/PI staining, (**d**) Intracellular ROS levels in hPDL-MSCs, represented as fluorescence intensity (%), following treatment with selected concentrations of H_2_O_2_ with or without postbiotics, assessed by flow cytometry. Data are expressed as mean ± SD (*n* = 3). Statistical significance is denoted as follows: ** *p* < 0.01, **** *p* < 0.0001, n.s: non-significant

To induce oxidative stress in hPDL-MSCs, a concentration of 300 µM H_2_O_2_ was selected, which reduced the viability to 50.69 ± 1.91% (Fig. 4a and c). Notably, postbiotic treatment significantly (*p* < 0.0001) improved the viability of H_2_O_2_-induced hPDL-MSCs compared to untreated H_2_O_2_-induced cells, as observed through both MTT and flow cytometry analyses (Fig. 4b and c). The effects of postbiotics on H_2_O_2_-induced hPDL-MSCs were further assessed by flow cytometry using DCFDA dye to measure intracellular ROS levels. The results showed that treatment with 300 µM H_2_O_2_ led to a significant increase in intracellular ROS levels (5.23-fold, *p* < 0.0001). However, postbiotic treatment for 24 h resulted in a 54.53 ± 2.01% reduction in intracellular ROS levels compared to untreated H_2_O_2_-induced hPDL-MSCs (*p* < 0.0001). Notably, postbiotic treatment alone did not significantly affect the intracellular ROS levels in hPDL-MSCs (Fig. 4d).

### Production of pro- and anti-inflammatory mediators

**Fig. 5:**
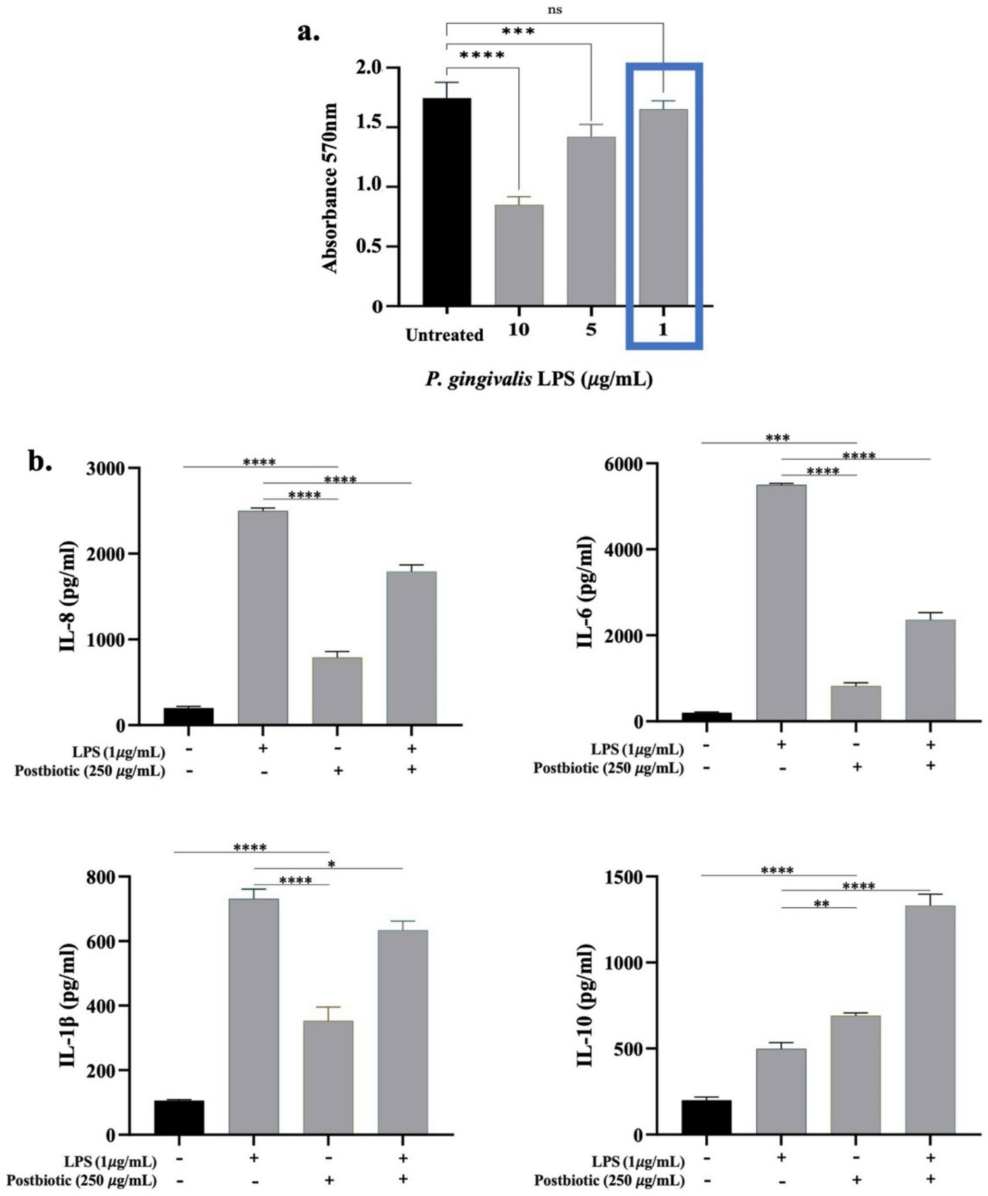
Immunomodulatory effects of postbiotics. (**a**) Determination of the lowest non-cytotoxic concentration of LPS using the MTT assay, (**b**) Levels of the *IL-8*, *IL-6*, *IL-1β* and *IL-10* in LPS-induced hPDL-MSCs treated with or without postbiotics. Data are expressed as mean ± SD (*n* = 3). Statistical significance is denoted as follows: *** *p* < 0.001, **** *p* < 0.0001, n.s: non-significant

The anti-inflammatory potential of postbiotics was further evaluated using LPS-induced hPDL-MSCs. To determine a non-cytotoxic concentration of LPS for hPDL-MSCs, an MTT assay was performed. The results showed that the lowest concentration of LPS (1 µg/mL) did not significantly affect the viability of hPDL-MSCs and was therefore selected for subsequent experiments (Fig. 5a). The effect of postbiotics on the production of IL-8, IL-6, IL-1β and IL-10 involved in the inflammatory and anti-inflammatory response, was assessed by ELISA analysis. The results indicated that LPS, acting as a pro-inflammatory stimulus, significantly increased the production of IL-8 (12.5-fold, *p* < 0.0001), IL-6 (27.5-fold, *p* < 0.001), and IL-1β (6.91-fold, *p* < 0.0001) in untreated hPDL-MSCs. Conversely, treatment with postbiotics markedly attenuated the levels of these pro-inflammatory cytokines in LPS-stimulated hPDL-MSCs. Specifically, postbiotics significantly reduced IL-8 levels by 1.36-fold compared to the elevated secretion of IL-8 in untreated LPS-stimulated hPDL-MSCs (*p* < 0.0001). Similarly, production of IL-6 and IL-1β were also reduced by 2.33-fold and 1.15-fold, respectively, in postbiotic-treated cells relative to untreated LPS-stimulated hPDL-MSCs. Notably, postbiotics significantly enhanced the production of IL-10, an anti-inflammatory cytokine, in LPS-induced hPDL-MSCs (2.67-fold, *p* < 0.0001) (Fig. 5b).

### Enhancement of collagen deposition

**Fig. 6:**
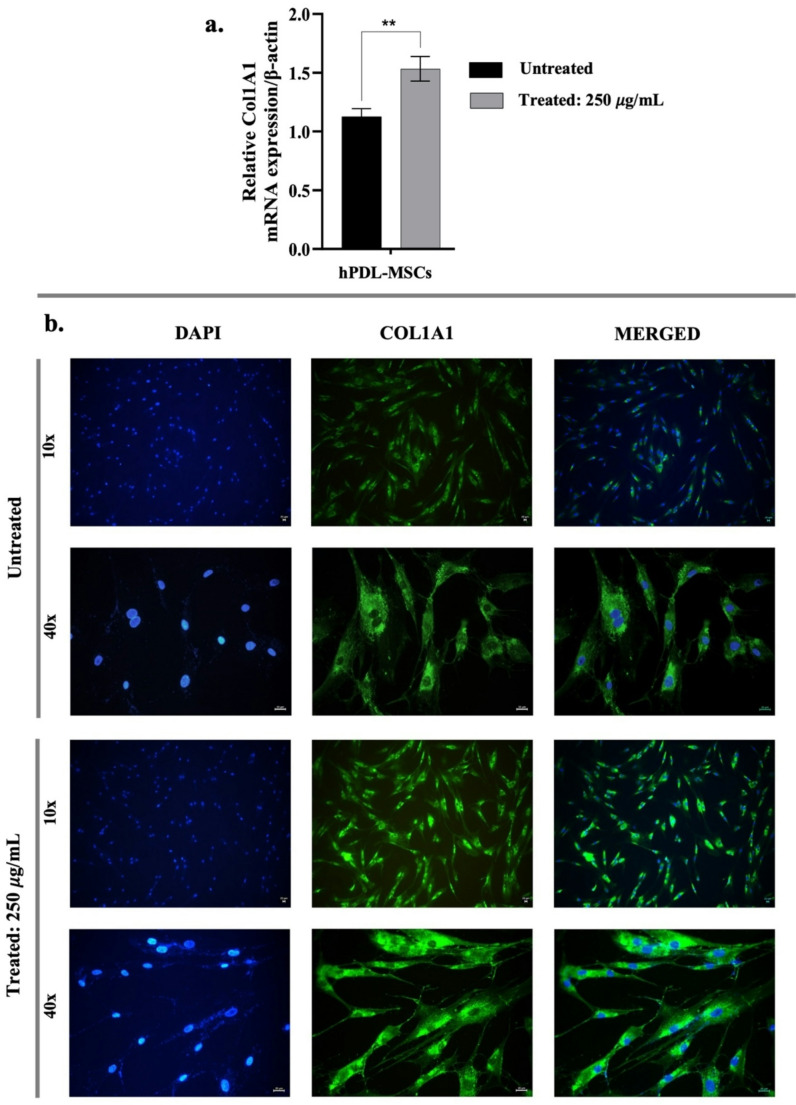
Collagen type 1 (COL1A1) production in hPDL-MSCs treated with postbiotics. (**a**) Gene expression levels assessed via qRT-PCR analysis, (**b**) Immunofluorescence detection visualized using fluorescence microscopy (COL1A1, green fluorescence; cellular nuclei: DAPI-stained, blue fluorescence). Data are expressed as mean ± SD (*n* = 3). Statistical significance is denoted as follows: ** *p* < 0.01

The effects of postbiotics on type I collagen synthesis in hPDL-MSCs were evaluated using qRT-PCR and immunofluorescence staining. Treatment with 250 µg/mL of postbiotics for 24 h significantly upregulated the COL1A1 mRNA expression by 1.36-fold (*p* < 0.01) compared to untreated cells (Fig. 6a). Furthermore, Furthermore, immunofluorescence analysis demonstrated increased COL1A1 production in hPDL-MSCs treated with postbiotics, as shown in Fig. 6b. These findings confirm that postbiotic treatment enhances type I collagen production in hPDL-MSCs.

## Discussion

Periodontal defects caused by periodontitis or trauma continue to pose significant challenges in the field of dentistry. The structural and biological integrity of the PDL, which plays an essential role in maintaining periodontal health, is critical for the long-term prognosis of periodontal tissues. The primary objective of effective periodontal treatment is to fully restore all components of the periodontium to their original architecture and functionality [34]. However, the limitations of conventional restoration methods in achieving functional periodontal tissue regeneration underscore the urgent need for innovative strategies. In recent years, tissue engineering, a multidisciplinary approach that integrates cells, biomaterials, and bioactive factors, has emerged as a tremendous future in addressing these challenges [17]. Among these approaches, hPDL-MSCs-based therapies hold great potential for achieving functional PDL restoration. These multipotent cells are characterized by minimal criteria, including plastic adherence, and in vitro trilineage differentiation to osteoblasts, adipocytes, and chondrocytes. Furthermore, when cultured, hPDL-MSCs exhibit the expression of specific surface markers such as CD73, CD90 and CD105, while lacking the expression of hematopoietic markers (CD34 and CD45) and the endothelial marker CD31 [14]. Within the scope of this study, hPDL-MSCs were isolated from healthy wisdom teeth and their immunophenotyping analysis confirmed the expression of characteristic surface markers, thereby validating their identity as hPDL-MSCs.

Recently, the application of hPDL-MSCs in tissue repair and regeneration has consistently attracted the attention of researchers. However, a major limitation in the clinical use of hPDL-MSCs for therapeutic purposes is the insufficient cell populations capable of migrating to the injury site and proliferating effectively. To address these limitations, various cytokines, growth factors, chemicals, and natural compounds have been explored, either individually or in combination, as culture media additives [17]. In this study, we propose a novel approach combining postbiotics with hPDL-MSCs and emphasize the existing gaps in their application for periodontal regeneration. To date, experimental evidence has demonstrated that probiotics contribute to tissue repair by secreting postbiotics [25, 35–37]. Although substantial evidence highlights the beneficial roles of postbiotics, neither their exact role on the dental stem cell proliferation nor the relevance of regulation of the periodontium remains unclear. Therefore, the current study aims to investigate the regenerative and protective effects of postbiotics derived from *L. plantarum* EIR/IF-1 strain on hPDL-MSCs in the context of periodontal diseases. Our findings revealed that postbiotics, at a concentration of 250 µg/mL, significantly enhanced the proliferation and migration of hPDL-MSCs. Furthermore, postbiotics enhanced cellular proliferation, thereby promoting the production of new collagen fibers by hPDL-MSCs. Consistent with our findings, Wu et al. [38] recently reported that postbiotics from *L. reuteri* stimulated the proliferation of human-induced pluripotent stem cells (h-iPSC) at lower doses but inhibited their proliferation at higher concentrations. They further demonstrated that sodium butyrate, a well-characterized bacterial metabolite and short-chain fatty acid present in postbiotics, exhibited similar effects on h-iPSC proliferation. Additionally, other recent studies have highlighted that *Lactobacillus*-derived postbiotics, such as lactate, enhance cell migration and tissue repair by promoting cellular proliferation [39]. Similarly, Neiva et al. [40] observed that B-vitamin complex supplementation accelerated wound healing following periodontal flap surgery. Based on this cumulative evidence, the primary components of postbiotics derived from *L. plantarum* EIR/IF-1, comprising lactate, stearic acid (C18:0), palmitic acid (C16:0), and B-complex vitamins (B1 thiamine, B5 pantothenic acid, and B12 cobalamin) as determined in our previous study [27], may contribute to their observed mechanism of action. However, further studies are warranted to elucidate the detailed effects of postbiotics on periodontal healing.

Although regenerative periodontal therapy aims to reconstruct periodontal supporting tissues [41], repairing lesions in this region remains a significant challenge due to the intricate structure of periodontal tissue, the surrounding inflammatory microenvironment, and oxidative damage [42]. In this context, postbiotics with their immunomodulatory and antioxidant properties, offer potential for maintaining periodontal tissue homeostasis. From this point of view, our findings demonstrated that postbiotics from *L. plantarum* EIR/IF-1 strain increased IL-10 levels, while decreasing IL-8, IL-1β and IL-6 levels in comparison to *P. gingivalis* LPS, thereby mitigating periodontal inflammation. To date, numerous studies have demonstrated the immunomodulatory effects of postbiotics through the regulation of cytokine production [43–45]. Consistent with our results, postbiotics derived from *L. plantarum* PD18 exhibited immunomodulatory properties in *P. gingivalis* LPS-induced cells, as evidenced by increased IL-10 levels alongside reduced IL-6 and IL-8 levels [46]. Additionally, recent studies have shown that postbiotics from the *L. curvatus* MG5246 strain downregulated IL-6 expression, a major pro-inflammatory cytokine, in human gingival fibroblast cells and demonstrated immunomodulatory effects in an in vivo mouse model [47].

Considering the close relationship between the various autoimmune or inflammatory oral conditions and cellular degeneration due to increased oxidative stress induced by oxygen-derived free radicals, maintaining homeostasis between oxidative and antioxidant mechanisms is critical for periodontal health [48]. Beyond their role in immune modulation, postbiotics derived from the *L. plantarum* EIR/IF-1 strain also act as a natural potent antioxidant. Similar to our results, postbiotics from *L. brevis* BK3, isolated from Kimchi, a traditional Korean fermented food, have been shown to protect fibroblast cells from oxidative stress induced by H₂O₂ and to reduce intracellular ROS production [49]. Moreover, several studies have also demonstrated that postbiotics as metabolic byproducts of probiotics, such as short-chain fatty acids, and vitamins, may mitigate oxidative damage and reduce the inflammatory burden in periodontal regeneration by modulating signaling pathways, including the Nrf2 pathway [48, 50]. Aligned with these findings, a study by Huang et al. [51] revealed that small-molecule peptides in postbiotics derived from *L. plantarum* SCS2 activate the Keap1-Nrf2 signaling pathway, thereby alleviating oxidative damage in H₂O₂-induced rat insulinoma cells. Additionally, postbiotics from *L. reuteri* SJ-47 contribute to antioxidant defense mechanisms by reducing ROS levels and upregulating antioxidant enzyme expression, ultimately exerting a protective effect on human fibroblasts under oxidative stress [52].

Since periodontal diseases are non-communicable and closely associated with an unbalanced oral microbiota, the application of microbial modulators, including postbiotics, shows promising beneficial effects and warrants further research [53]. Based on studies conducted on patients, lozenges or tablets containing postbiotics have been shown to reduce caries incidence in preschool and schoolchildren compared to standard preventive care. For adults with periodontitis, the adjunctive use of synbiotic and postbiotic products appears to enhance the outcome of conventional scaling and root planing [53]. In another study, a soothing gel containing postbiotics (*Lactobacillus* Ferment) was found to be a valuable adjunct to non-surgical periodontal treatment, improving periodontal health in patients with Down syndrome and reducing BOP (Bleeding on Probing) after 6 months of treatment [54]. Basir et al. [55] investigated the effect of postbiotic toothpaste (*Bifidobacterium animalis* subsp. *animalis*) on salivary levels of Immunoglobulin A (IgA) and pH in children in a triple-blind, randomized, placebo-controlled trial. According to the clinical study results, the administration of postbiotics increased oral immunity in children. A recent study also showed that postbiotics co-treatment with dental fillings could improve prognosis for patients with dental caries compared to dental fillings alone [56]. All these results suggest that postbiotics could have a significant role in clinical applications. Therefore, our findings provide a more comprehensive understanding of the therapeutic potential of postbiotics in the context of periodontal regeneration and support their integration into dental product such as toothpaste, mouthwash, scaffolds and gels, as a natural, cost-effective, novel, and safe bio-material. The successful application of postbiotic preparations in the clinical setting can provide crucial support and greater assurance for oral health. However, several questions remain, including the dose-response effects, the most effective mode of administration, and suitable combinations with prebiotics and probiotics [53]. Investigating the molecular mechanisms underlying the immune-modulatory and antioxidant effects of postbiotics is also critical for advancing their clinical application in regenerative medicine. Another knowledge gap is the lack of pre-clinical and clinical studies, which would be essential to assess the sustained effects of postbiotics in periodontal regeneration.

## Conclusion

In the present study, we found consistent evidence that postbiotics derived from the *L. plantarum* EIR/IF-1 exhibit significant biological activity throughout all stages of the healing process, ranging from the modulation of initial inflammatory response to LPS stimulation, followed by the promotion of cell migration, proliferation, and collagen synthesis, ultimately improving long-term regenerative potential. Future studies should focus on improving clinical outcomes and enhancing patient quality of life in the fields of oral medicine and dentistry.

## Electronic supplementary material

Below is the link to the electronic supplementary material.

Supplementary Material 1

## Data Availability

Data is provided within the manuscript.

## References

[1] Yin J, Li Y, Feng M, Li L (2022) Understanding the feelings and experiences of patients with periodontal disease: A qualitative meta-synthesis. Health Qual Life Outcome 20(1):126. 10.1186/s12955-022-02042-5 10.1186/s12955-022-02042-5 PMC9419312 36028888

[2] Caton JG, Armitage G, Berglundh T, Chapple ILC, Jepsen S et al (2018) A new classification scheme for periodontal and peri-implant diseases and conditions—introduction and key changes from the 1999 classification. J Clin Periodontol 45:1–8. 10.1111/jcpe.12935 10.1111/jcpe.12935 29926489

[3] Eke PI, Borgnakke WS, Genco RJ (2020) Recent epidemiologic trends in periodontitis in the USA. Periodontol 82(1):257–267. 10.1111/prd.12323 10.1111/prd.12323 31850640

[4] Chen MX, Zhong YJ, Dong QQ, Wong HM, Wen YF (2021) Global, regional, and National burden of severe periodontitis, 1990–2019: an analysis of the global burden of disease study 2019. J Clin Periodontol 48(9):1165–1188. 10.1111/jcpe.13506 34101223 10.1111/jcpe.13506

[5] Guo H, Bai X, Wang X et al (2022) Development and regeneration of periodontal supporting tissues. Genesis 60(8–9):e23491. 10.1002/dvg.23491 35785409 10.1002/dvg.23491

[6] Shaikh MS, Zafar MS, Alnazzawi A (2021) Comparing nanohydroxyapatite graft and other bone grafts in the repair of periodontal infrabony lesions: asystematic review and meta-analysis. Int J Mol Sci 22(21):12021. 10.3390/ijms222112021 34769451 10.3390/ijms222112021 PMC8584357

[7] Reynolds MA, Kao RT, Camargo PM, Caton JG, Clem DS et al (2015) Periodontal regeneration - intrabony defects: A consensus report from the AAP regeneration workshop. J Periodontol 86(2):105–107. 10.1902/jop.2015.140378 10.1902/jop.2015.140378 25315019

[8] Ferrarotti F, Romano F, Gamba MN, Quirico A, Giraudi M et al (2018) Human intrabony defect regeneration with micrografts containing dental pulp stem cells: A randomized controlled clinical trial. J Clin Periodontol 45(7):841–850. 10.1111/jcpe.12931 29779220 10.1111/jcpe.12931

[9] Chen FM, Gao LN, Tian BM, Zhang XY, Zhang YJ et al (2016) Treatment of periodontal intrabony defects using autologous periodontal ligament stem cells: A randomized clinical trial. Stem Cell Res Ther 19(7):33. 10.1186/s13287-016-0288-1 10.1186/s13287-016-0288-1 PMC4761216 26895633

[10] Sun L, Du X, Kuang H et al (2023) Stem cell-based therapy in periodontal regeneration: a systematic review and meta-analysis of clinical studies. BMC Oral Health 23:492. 10.1186/s12903-023-03186-6 37454056 10.1186/s12903-023-03186-6 PMC10350264

[11] Sagaradze GD, Basalova NA, Efimenko AY, Tkachuk VA (2020) Mesenchymal stromal cells as critical contributors to tissue regeneration. Front Cell Dev Biol 8:576176. 10.3389/fcell.2020.576176 33102483 10.3389/fcell.2020.576176 PMC7546871

[12] Almeida-Porada G, Atala AJ, Porada CD (2020) Therapeutic mesenchymal stromal cells for immunotherapy and for gene and drug delivery. Mol Ther Methods Clin Dev 16:204–224. 10.1016/j.omtm.2020.01.005 32071924 10.1016/j.omtm.2020.01.005 PMC7012781

[13] Huang F, Thokerunga E, He F, Zhu X, Wang Zi Tu J (2022) Research progress of the application of mesenchymal stem cells in chronic inflammatory systemic diseases. Stem Cell Res Ther 13:1. 10.1186/s13287-021-02613-1 34998430 10.1186/s13287-021-02613-1 PMC8742935

[14] Miłek O, Schwarz K, Miletić A, Reisinger J, Kovar A, Behm C, Andrukhov O (2025) Regulation and functional importance of human periodontal ligament mesenchymal stromal cells with various rates of CD146 + cells. Front Cell Dev Biol 13:1532898. 10.3389/fcell.2025.1532898 40123853 10.3389/fcell.2025.1532898 PMC11925893

[15] Behm C, Miłek O, Rausch-Fan X et al (2024) Paracrine- and cell-contact-mediated Immunomodulatory effects of human periodontal ligament-derived mesenchymal stromal cells on CD4 + T lymphocytes. Stem Cell Res Ther 15:154. 10.1186/s13287-024-03759-4 38816862 10.1186/s13287-024-03759-4 PMC11141051

[16] Queiroz A, Albuquerque-Souza E, Gasparoni LM et al (2021) Therapeutic potential of periodontal ligament stem cells. World J Stem Cells 13(6):605–618. 10.4252/wjsc.v13.i6.605 34249230 10.4252/wjsc.v13.i6.605 PMC8246246

[17] Di Vito A, Bria J, Antonelli A, Mesuraca M, Barni T et al (2023) A review of novel strategies for human periodontal ligament stem cell *ex vivo* expansion: are they an evidence-based promise for regenerative periodontal therapy? Int J Mol Sci 24(9):7798. 10.3390/ijms24097798 37175504 10.3390/ijms24097798 PMC10178011

[18] Santacroce L, Passarelli PC, Azzolino D et al (2023) Oral microbiota in human health and disease: A perspective. Exp Biol Med (Maywood) 248(15):1288–1301. 10.1177/15353702231187645 37688509 10.1177/15353702231187645 PMC10625343

[19] Baddouri L, Hannig M (2024) Probiotics as an adjunctive therapy in periodontitis treatment—reality or illusion—a clinical perspective. NPJ Biofilm Microbiom 10:148. 10.1038/s41522-024-00614-5 10.1038/s41522-024-00614-5 PMC11649906 39681550

[20] Yu Z, Chen J, Liu Y et al (2023) The role of potential probiotic strains *Lactobacillus reuteri *in various intestinal diseases: new roles for an old player. Front Microbiol 14:1095555. 10.3389/fmicb.2023.1095555 36819028 10.3389/fmicb.2023.1095555 PMC9932687

[21] How YH, Yeo SK (2021) Oral probiotic and its delivery carriers to improve oral health: A review. Microbiol 167(8). 10.1099/mic.0.001076 10.1099/mic.0.001076 34351255

[22] Prajapati K, Bisani K, Prajapati H et al (2024) Advances in probiotics research: mechanisms of action, health benefits, and limitations in applications. Syst Microbiol Biomanuf 4:386–406. 10.1007/s43393-023-00208-w

[23] Adams CA (2010) The probiotic paradox: live and dead cells are biological response modifiers. Nutr Res Rev 23:37–46. 10.1017/S0954422410000090 20403231 10.1017/S0954422410000090

[24] Salminen S, Collado MC, Endo A et al (2021) The international scientific association of probiotics and prebiotics (ISAPP) consensus statement on the definition and scope of postbiotics. Nat Rev Gastroenterol Hepatol 18:649–667. 10.1038/s41575-021-00440-6 33948025 10.1038/s41575-021-00440-6 PMC8387231

[25] Liang B, Xing D (2023) The current and future perspectives of postbiotics. Probiotics Antimicro Prot 15:1626–1643. 10.1007/s12602-023-10045-x 10.1007/s12602-023-10045-x PMC9913028 36763279

[26] Wei X, Qian S, Yang Y, Mo J (2024) Microbiome-based therapies for periodontitis and peri-implantitis. Oral Dis 30(5):2838–2857. 10.1111/odi.14782 37890080 10.1111/odi.14782

[27] OmerOglou E, Karaca B, Kibar H, Haliscelik O, Kiran F (2022) The role of microbiota-derived postbiotic mediators on biofilm formation and quorum sensing-mediated virulence of *Streptococcus mutans*: A perspective on preventing dental caries. Microb Pathog 164:105390. 10.1016/j.micpath.2022.105390 35092835 10.1016/j.micpath.2022.105390

[28] Karaca B, Gursoy M, Kiran F, Loimaranta V, Söderling E, Gursoy UK (2023) Postbiotics of the *Lactiplantibacillus plantarum* EIR/IF-1 strain show antimicrobial activity against oral microorganisms with pH adaptation capability. Microbiol Res 14(3):1442–1456. 10.3390/microbiolres14030098 10.3390/microorganisms10112200 PMC9693231 36363792

[29] Karaca B, Haliscelik O, Gursoy M, Kiran F, Loimaranta V et al (2022) Analysis of chemical structure and antibiofilm properties of exopolysaccharides from *Lactiplantibacillus plantarum* EIR/IF-1 postbiotics. Microorganisms 10(11):2200. 10.3390/microorganisms10112200 36363792 10.3390/microorganisms10112200 PMC9693231

[30] Park JC, Kim JM, Jung IH, Kim JC, Choi SH et al (2011) Isolation and characterization of human periodontal ligament (PDL) stem cells (PDLSCs) from the inflamed PDL tissue: *in vitro* and *in vivo* evaluations. J Clin Periodontol 38(8):721–731. 10.1111/j.1600-051X.2011.01716.x 21449989 10.1111/j.1600-051X.2011.01716.x

[31] Mosmann T (1983) Rapid colorimetric assay for cellular growth and survival: application to proliferation and cytotoxicity assays. J Immunol Methods 65:55–63. 10.1016/0022-1759(83)90303-4 6606682 10.1016/0022-1759(83)90303-4

[32] Liang CC, Park A, Guan JL (2007) *In vitro* scratch assay: A convenient and inexpensive method for analysis of cell migration *in vitro*. Nat Protoc 2:329–333. 10.1038/nprot.2007.30 17406593 10.1038/nprot.2007.30

[33] Liu J, Chen S, Ren W et al (2017) Lipopolysaccharide-induced suppression of periodontal ligament cell proliferation and apoptosis are strengthened under high glucose conditions. Arch Oral Biol 79:70–76. 10.1016/j.archoralbio.2017.01.007 28327438 10.1016/j.archoralbio.2017.01.007

[34] Huang TH, Chen JY, Suo WH, Shao WR et al (2024) Unlocking the future of periodontal regeneration: an interdisciplinary approach to tissue engineering and advanced therapeutics. Biomedicines 12(5):1090. 10.3390/biomedicines12051090 38791052 10.3390/biomedicines12051090 PMC11118048

[35] Badaluta VA, Curuțiu C, Dițu LM, Holban AM, Lazar V (2024) Probiotics in wound healing. Int J Mol Sci 25(11):5723. 10.3390/ijms25115723 38891909 10.3390/ijms25115723 PMC11171735

[36] Lukic J, Chen V, Strahinic I et al (2017) Probiotics or pro-healers: the role of beneficial bacteria in tissue repair. Wound Repair Regen 25(6):912–922. 10.1111/wrr.12607 29315980 10.1111/wrr.12607 PMC5854537

[37] Park JH, Kotani T, Konno T et al (2016) Promotion of intestinal epithelial cell turnover by commensal bacteria: role of short-chain fatty acids. PLoS ONE 11(5):e0156334. 10.1371/journal.pone.0156334 27232601 10.1371/journal.pone.0156334 PMC4883796

[38] Wu D, Fu R, Scoffield J, Kannappan R (2018) Probiotic metabolites promote stem cell proliferation and differentiation. Faseb J 32. 10.1096/fasebj.2018.32.1_supplement.615.4.:615.4

[39] Yu Y, Yang W, Bilotta AJ, Zhao X, Cong Y, Li Y (2021) L-lactate promotes intestinal epithelial cell migration to inhibit colitis. FASEB J 35(4):e21554. 10.1096/fj.202100095R 33742715 10.1096/fj.202100095R

[40] Neiva RF, Al-Shammari K, Nociti FH, Soehren S, Wang H (2005) Effects of vitamin-B complex supplementation on periodontal wound healing. J Periodontol 76:1084–1091. 10.1902/jop.2005.76.7.1084 16018750 10.1902/jop.2005.76.7.1084

[41] Goriuc A, Foia L, Cojocaru K, Diaconu-Popa D et al (2023) The role and involvement of stem cells in periodontology. Biomedicines 11(2):387. 10.3390/biomedicines11020387 36830924 10.3390/biomedicines11020387 PMC9953576

[42] Liu A-Q, Hu C-H, Jin F, Zhang L-S, Xuan K (2018) Contributions of bioactive molecules in stem cell-based periodontal regeneration. Int J Mol Sci 19(4):1016. 10.3390/ijms19041016 29597317 10.3390/ijms19041016 PMC5979460

[43] Arasu KA, Rajasekar T (2024) Immunomodulatory activity of postbiotics from *Lactobacillus*. In: Dharumadurai D (ed) Postbiotics. Methods and protocols in food science. Humana, New York, NY. 10.1007/978-1-0716-3421-9_25

[44] Szydłowska A, Sionek B (2022) Probiotics and postbiotics as the functional food components affecting the immune response. Microorganisms 11(1):104. 10.3390/microorganisms11010104 36677396 10.3390/microorganisms11010104 PMC9862734

[45] Roberts KD, Ahmed S, San Valentin E et al (2024) Immunomodulatory properties of multi-strain postbiotics on human CD14 + monocytes. Life (Basel) 14(12):1673. 10.3390/life14121673 39768380 10.3390/life14121673 PMC11728152

[46] Butrungrod W, Chaiyasut C, Makhamrueang N et al (2024) Postbiotic metabolite derived from *Lactiplantibacillus plantarum* PD18 maintains the integrity of cell barriers and affects biomarkers associated with periodontal disease. Antibiot (Basel) 13(11):1054. 10.3390/antibiotics13111054 10.3390/antibiotics13111054 PMC11591352 39596748

[47] Jung JI, Kim YG, Kang CH, Imm JY (2021) Effects of *Lactobacillus curvatus* MG5246 on inflammatory markers in *Porphyromonas gingivalis* lipopolysaccharide-sensitized human gingival fibroblasts and periodontitis rat model. Food Sci Biotechnol 31(1):111–120. 10.1007/s10068-021-01009-4 35059235 10.1007/s10068-021-01009-4 PMC8733125

[48] Karaca B, Yilmaz M, Gursoy UK (2022) Targeting Nrf2 with probiotics and postbiotics in the treatment of periodontitis. Biomol 12(5):729. 10.3390/biom12050729 10.3390/biom12050729 PMC9139160 35625655

[49] Lee YS, Lee SJ, Jang WJ, Lee EW (2024) Protective effects of the postbiotic *Levilactobacillus brevis* BK3 against H_2_O_2_-induced oxidative damage in skin cells. J Microbiol Biotechnol 34(7):1401–1409. 10.4014/jmb.2403.03010 38881180 10.4014/jmb.2403.03010 PMC11294649

[50] Rezaie N, Aghamohammad S, Haj Agha Gholizadeh Khiavi E et al (2024) The comparative anti-oxidant and anti-inflammatory efficacy of postbiotics and probiotics through Nrf-2 and NF-kB pathways in DSS-induced colitis model. Sci Rep 14:11560. 10.1038/s41598-024-62441-0 38773299 10.1038/s41598-024-62441-0 PMC11109304

[51] Huang X, Jiang L, Zhang Y, Jing L, Meng X, Liu S (2024) Small-polecule peptides from *Lactiplantibacillus plantarum* SCS2 attenuate H_2_O_2_-induced oxidative damage in INS-1 cells via regulating the Keap1-Nrf2 signaling pathway. J Food Biochem 1(1):1–12. 10.1155/2024/8847548

[52] Zhao J, Fu H, Zhang Y, Li M, Wang D, Zhao D, Zhang J, Wang C (2022) Protective effects of *Lactobacillus reuteri* SJ-47 strain exopolysaccharides on human skin fibroblasts damaged by UVA radiation. Bioresour Bioprocess 9(1):127. 10.1186/s40643-022-00617-0 38647814 10.1186/s40643-022-00617-0 PMC10992028

[53] Twetman S, Belstrøm D (2025) Effect of synbiotic and postbiotic supplements on dental caries and periodontal diseases—A comprehensive review. Int J Environ Res Public Health 22:72. 10.3390/ijerph22010072 39857525 10.3390/ijerph22010072 PMC11764861

[54] Scribante A, Appendino P, Maiorani C et al (2025) Home efficacy of a postbiotic-based gel compared with a gel without active ingredients for the treatment of gingival inflammation in patients with down syndrome: A randomized controlled study. Dent J 13:62. 10.3390/dj13020062 10.3390/dj13020062 PMC11854621 39996936

[55] Basir L, Moghimipour E, Saadatzadeh A, Cheraghian B, Khanehmasjedi S (2022) Effect of postbiotic-toothpaste on salivary levels of IgA in 6- to 12-year-old children: Study protocol for a randomized triple-blind placebo-controlled trial. Front Pediatr 10:1042973. 10.3389/fped.2022.1042973 36578663 10.3389/fped.2022.1042973 PMC9790979

[56] Liu Q, Ma T, Feng C et al (2024) Adjuvant postbiotic administration improves dental caries prognosis by restoring the oral microbiota. Food Sci Hum Well 13(5):2690–2702. 10.26599/FSHW.2022.9250217

