# Supplementary figures and images for: Evaluation of the anti-inflammatory, antioxidant and regenerative effects of microbiota-derived postbiotics in human periodontal ligament mesenchymal stromal cells
Source: Clin Oral Investig. 2025 Apr 23;29(5):262. doi: 10.1007/s00784-025-06341-1 (PMC12014813; doi:10.1007/s00784-025-06341-1)

**Supplementary Fig. 1**

**
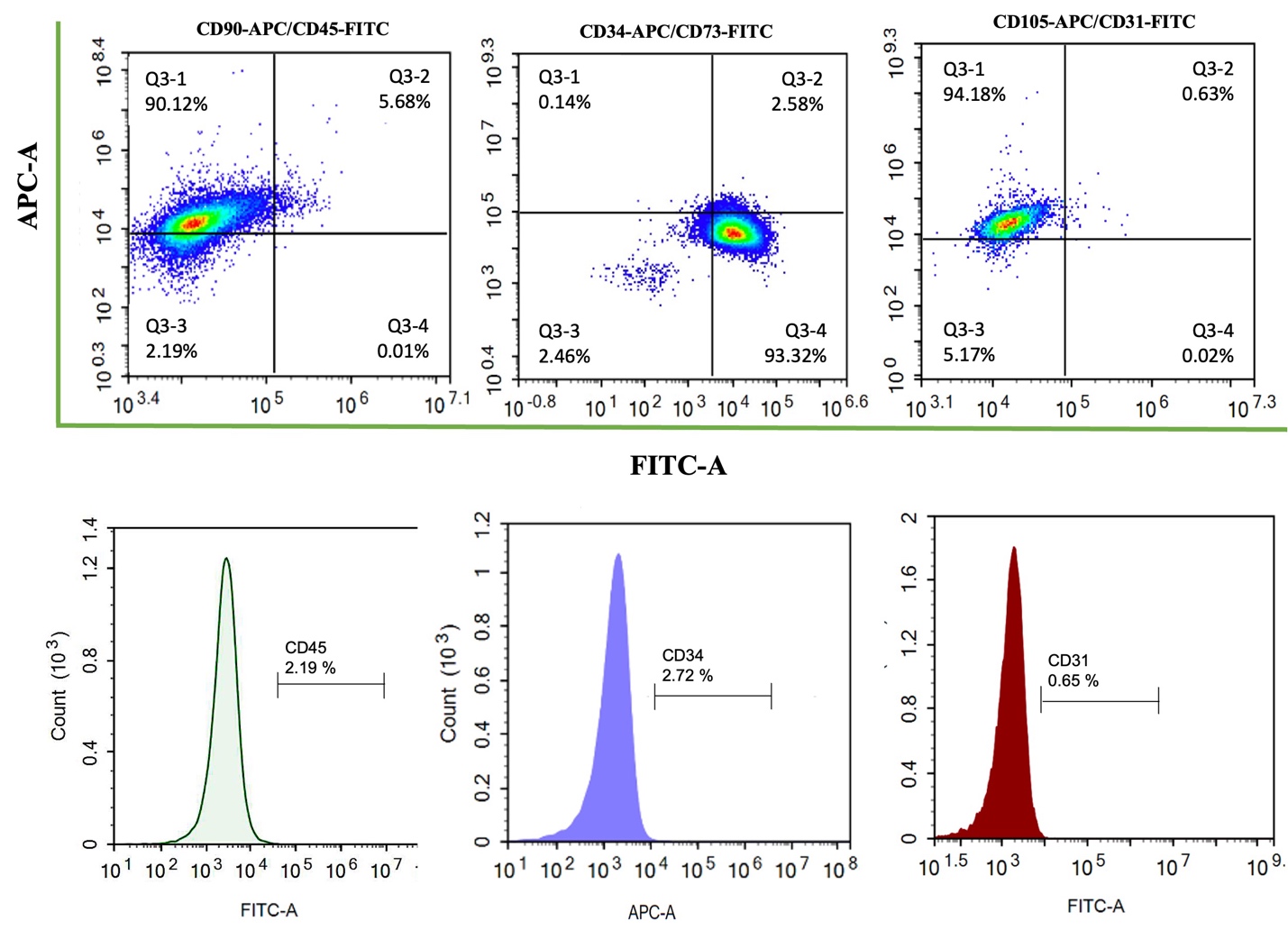
**

**Fig.1** Immunophenotypic analysis of hPDL-MSCs using flow cytometry.

Supplement: Supplementary file 1 — Supplementary Material 1 [file 784_2025_6341_MOESM1_ESM.docx]
